# Next Generation Sequencing Uncovers Unexpected Bacterial Pathogens in Ticks in Western Europe

**DOI:** 10.1371/journal.pone.0081439

**Published:** 2013-11-27

**Authors:** Muriel Vayssier-Taussat, Sara Moutailler, Lorraine Michelet, Elodie Devillers, Sarah Bonnet, Justine Cheval, Charles Hébert, Marc Eloit

**Affiliations:** 1 USC Bipar, INRA, Anses, Maisons-Alfort, France; 2 PathoQuest SAS, Paris, France; 3 Ecole Nationale Vétérinaire d′Alfort, UMR 1161 Virologie ENVA, INRA, ANSES, Maisons-Alfort, France; 4 Institut Pasteur, Laboratory of Pathogen Discovery, Paris, France; University of Kentucky College of Medicine, United States of America

## Abstract

**Background and Aims:**

Ticks are highly susceptible to global environmental and socio-economical changes. Several tick-borne pathogens have been reported in new geographical regions while new species, strains or genetic variants of tick-borne microorganisms are continually being detected. However, tick-borne pathogens are still poorly understood, and it is estimated that half of all human tick-borne disease has an unknown origin. Therefore in order to prevent these diseases, more effort is required to identify unknown or unexpected tick-borne pathogens. *Ixodes ricinus* is the vector for a broad range of bacterial pathogens and the most prevalent tick in Europe. The aim of the present study was to evaluate the capability of Next Generation Sequencing (NGS) to extend the inventory of pathogenic bacteria carried by this species of tick in France.

**Methods:**

RNA and DNA were extracted from 1450 *I. ricinus* questing nymphs collected by flagging in Alsace, France. RNA was pooled and used for NGS. Following *de novo* assembly, bacterial contigs were assigned to the closest known taxonomy. DNA was used for real time PCR to confirm taxonomic species assignment of NGS-derived contigs for the doubtful cases, and for determination of prevalence.

**Results:**

We have generated a global in-depth picture of tick-borne bacteria. We identified RNA from the main pathogenic bacterial species known to be transmitted by *I. ricinus*. In addition we also identified unanticipated bacterial species for which we have estimated the prevalence within those ticks inhabiting the studied areas.

**Conclusions:**

The data obtained from this study has proven that NGS has an enormous potential to detect the unexpected and provides the means to monitor pathogen occurrence.

## Introduction

Ticks are highly susceptible to global environmental and socio-economical changes [1], such that the incidence of tick-borne disease is increasing worldwide [2], and that some tick-borne pathogens have been reported in new geographical regions [3], [4]. *Ixodes ricinus* is the most common European tick species, and is the vector for a broad range of bacterial pathogens [2], [5].

Due to recent advances in molecular biology, new species, strains or genetic variants of microorganisms are continually being detected in ticks, resulting in an ever-increasing list of pathogens capable of infecting livestock and humans. In Europeans, the most prevalent tick-borne human disease is Lyme borreliosis, caused by any of four different *Borrelia* species; *B. burgdorferii* sensu stricto, *B. afzelii*, *B. garini*, and *B. spielmanii* [6], with over 50 000 new cases each year in Europe [7]. Lyme disease has a high rate of diagnosis, mainly due to its distinctive symptoms and the existence of effective serological tests. Although, in addition to these typical cases, patients with tick bites can also be infected by many other bacteria including *Anaplasma phagocytophilum*, *Rickettsiae* spp., “*Candidatus* Neoehrlichia mikurensis”, *Ehrlichia* spp., *Bartonella* spp. and *Francisella* spp. Some of these species have been identified in ticks decades prior to their association with human disease, whereas others have only been discovered very recently. Therefore we can confidently assume that many tick-associated pathogens are still unknown [3], [4], [8]. In cases of co-infection, symptoms induced by these microorganisms are often non-specific and can be aggravated by pre-existing or concurrent diseases [9] making diagnosis exceedingly difficult. It is estimated that up to half of human tick-borne disease has an unknown microbiological origin [10]. Consequently, in order to identify novel or unexpected tick-borne pathogens in their natural hosts, concerted efforts are required to develop efficient detection methods.

Up until now, most studies aiming to identify tick-borne pathogens in their vectors have only been able to assess minimal numbers of pathogens at a time, which is partly due to technological limitations. Indeed, complete screens of microorganisms in natural host populations (both reservoirs and vectors) were out of reach using standard laboratory procedures. Identification of microorganisms in biological samples has been dominated by the use of culture-dependent methods and by conventional molecular approaches. These methodologies suffer from major limitations: (i) isolation in cell culture or in synthetic medium is difficult for many microorganisms and (ii) molecular identification was mostly based on the use of specific primers combined with real-time PCR, which are only capable of detecting a selected number of species. However, the recent rapid development of Next Generation Sequencing (NGS) methods combined with bioinformatics has revolutionized the research field of epidemiology and diagnosis of infectious diseases, and has effectively overcome those limitations. NGS could be effective tools in diagnosing pathogens for medical and veterinary purposes [11]–[18] and these techniques has recently been successfully used to identify the bacterial communities associated with *I. ricinus* ticks [19], [20]. In these studies, the authors used the amplification and the sequencing of the hyper-variable region V6 of the 16S rRNA encoding genes. If this gene is well adapted to describe bacterial communities, the analysis of its sequence does not allow identifying bacteria at the species level. Ticks carry many bacterial species, including endogenous ones (symbionts and commensals). In ticks, symbionts are often phylogenetically close to pathogens and 16S rRNA diversity does not allow, for instance, distinguishing symbiotic *Rickettsia* species from pathogenic ones. Besides this problem, within a bacteria genus, many species exist. This is the case for B*artonella* sp., for which some species are known to be pathogenic for humans (or animals) and other have never been associated to diseases. Thus identifying them at the species level is very important. This is also the case for the bacteria belonging to the complex of *Borrelia burgdorferi* sensu lato, responsible of Lyme diseases, for which the analysis of16SrRNA diversity is not efficient to identify them at the species level.

In this context, we undertook a study to produce an inventory of predicted and/or unexpected pathogenic bacteria carried by ticks, in a geographical region with abundant ticks and a concomitant high risk of disease transmission. To overcome the limitation of deep sequencing of 16S RNA encoding genes, we sequenced the whole transcriptome of ticks and their resident microbiota.

## Materials and Methods

### Tick collection and extract preparation

No specific permits were required for the described field studies. These field studies did not involve endangered or protected species. A total of 1450 questing nymphs and 62 females of the *I. ricinus* tick were collected by flagging from three wooded habitats in Eastern France (Alsace Department, [Murbach (47°55′05”N, 7°8′46”E), Hohbuhl (48°27′33”N, 7°17′22”E) and Wasselonne (48°38′09”N, 7°21′45”E)]). All collected ticks were washed as previously described [21], nymphs were pooled into groups of 25 individuals (58 pools in total) and adults into groups of 2 individuals (31 pools in total) and crushed in 300 µl of Dulbecco's MEM (DMEM) medium supplemented by 10% fetal bovine serum. A pool of 15 *I. ricinus* nymphs from our pathogen-free colony was equivalently treated and used as a reference. This control colony originated from female ticks collected in Murbach (Alsace, France) and was bred as previously described [22]. The pathogen-free colony was obtained as follows; females from the field were engorged on rabbits and allowed to lay eggs. Female tick DNA samples were extracted and different PCRs were performed to test for the presence of *Borrelia* spp., *Bartonella* spp., *Anaplasma* spp., *Rickettsia* spp., *Francisella* spp. and *Coxiella* spp. Only larvae from “pathogen-free” female ticks were conserved and maintained in our colony.

### DNA and RNA extraction

Crushed tick pools were divided into two equal samples. Total RNA, used to identify viable and replicating microorganisms, and total DNA, for both pathogen taxonomic confirmation and prevalence studies, were then extracted from the nymphs using the Nucleospin RNA II kit (Macherey-Nagel, Duren, Germany), and the Wizard genomic DNA purification kit (Promega, Madison, WI, USA) following manufacturers' instructions.

### High throughput sequencing and data analysis

High throughput sequencing was only performed on nymphs, and all RNA samples were pooled before sequencing. RNA was first retro-transcribed to cDNA, then randomly amplified using the Multiple Displacement Amplification (MDA) protocol with phi29 polymerase and random hexamers as described [17]. Library preparations and sequencing with an Illumina HiSeq2000 sequencer were outsourced to DNAVision (Belgium). Wild and pathogen-free samples were sequenced to a depth of 100 Mio and 62 Mio paired-end reads of 101 bp respectively. Raw sequence reads were trimmed according to their quality score. At the time of analysis, there was no published reference genome for *I. ricinus* available in public databases, so sequences from the ticks themselves, or from symbiotic or commensal bacteria naturally found in ticks, were removed from the analysis by subtracting sequences derived from the pathogen-free reference sample using the SOAP2 aligner tool. Finally, *de novo* assembly was performed on all remaining reads (7.7 Mio), producing 174 841 contigs. Taxonomic assignation of these contigs was achieved via successive sequence alignment using nucleotide and protein databases and the BLAST algorithm. Contigs were assigned the closest known taxonomy according to their identity percentage, and distant alignments were not considered. Of the assigned reads, 12.22% of the cDNA derived sequences were of bacterial origin.

### Specific Real-Time PCR for taxonomic species assignment of NGS-derived contigs confirmation

Real-time PCR on DNA samples was performed to confirm taxonomic species assignment of NGS-derived contigs for those cases in doubt: *Francisella tularensis*, *Coxiella burnettii*, *Ehrlichia canis*, *Rickettsia felis* and *R. canadensis*. All DNA samples were pooled as for RNA analysis. Primers and probes used for amplification are described in Table 1.

**Table 1:** Primers and probes used for pathogen confirmation or prevalence estimation in ticks.

Pathogen	Target	Primers and Probe Sequence (5′ → 3′)	Amplicon size (bp)	Reference
*Borrelia miyamotoi*	glpQ	CACGACCCAGAAATTGACACA	94	This study
		GTGTGAAGTCAGTGGCGTAAT		
		TCGTCCGTTTTCTCTAGCTCGATTGGG		
*Coxiella burnetti*	idc	AGGCCCGTCCGTTATTTTACG	74	Adapted from [47]
		CGGAAAATCACCATATTCACCTT		
		TTCAGGCGTTTTGACCGGGCTTGGC		
*Ehrlichia canis*	dsb	AATACTTGGTGAGTCTTCACTCA	110	Adapted from [30]
		GTTGCTTGTAATGTAGTGCTGC		
		AAGTTGCCCAAGCAGCACTAGCTGTAC		
*Francisella tularensis*	tul4	ACCCACAAGGAAGTGTAAGATTA	76	Adapted from [48]
		GTAATTGGGAAGCTTGTATCATG		
		AATGGCAGGCTCCAGAAGGTTCTAAGT		
*Rickettsia felis*	orfB	CCCTTTTCGTAACGCTTTGCT	149	49
		GGGCTAAACCAGGGAAACCT		
		TGTTCCGGTTTTAACGGCAGATACCCA		
*Rickettsia canadensis*	ompA	AGTGGGACATTGGGTGTTGC	129	This study
		GGCAACACCTTGAGCATTATC		
		CTCCTGCCTGCGTTATACCATGCCA		
*Candidatus* Neoehrlichia mikurensis	groEL	AGAGACATCATTCGCATTTTGGA	96	This study
		TTCCGGTGTACCATAAGGCTT		
		AGATGCTGTTGGATGTACTGCTGGACC		

DNA pre-amplification was performed using the TaqMan PreAmp Master Mix kit (Applied Biosystems). Primers were pooled at a final concentration of 0.2X with equal primer concentrations (20 µM each). The reaction was performed in a final volume of 5 µl containing 2.5 µl TaqMan PreAmp Master Mix (2X), 1.2 µl of the pooled primer mix (0.2X) and 1.3 µl of DNA, with one cycle at 95°C for 10 min, 14 cycles at 95°C for 15 sec and 4 min at 60°C. After the pre-amplification PCR, products were diluted 1∶20 and stored at −20°C prior to use in qPCR. All fluorogenic probes were synthesized with a 6-carboxy-fluorescein (FAM) reporter molecule attached to the 5′ end and a Black Hole Quencher 1 (BHQ1) attached to the 3′ end. Real-time Taqman PCR assays were performed in a final volume of 12 µl using the LightCycler® 480 Probe Master mix (Roche Applied Science, Germany) at a 1X final concentration, with primers and probes at 200 nM and pre-amplified DNA. Thermal cycling conditions were as follows: 95°C for 5 min, 45 cycles at 95°C for 10 s and 60°C for 15 s and then 40°C for 10 s.

### Specific Real-Time PCR for prevalence of unexpected pathogens in ticks

Real-time PCR was performed to determine the prevalence of three unexpected tick-borne bacteria DNA in ticks: “*Candidatus* Neoehrlichia mikurensis”, *Borrelia miyamotoi*, and *R. felis*. We assessed 58 pools of 25 nymphs according to the collection location (corresponding to a total of 1450 nymphs) and 31 pools of 2 females (corresponding to a total of 62 females). The primers for amplification of “*Candidatus* Neoehrlichia mikurensis” and *Borrelia miyamotoi* were specifically designed for this study and are presented in Table 1. Real-time PCR was conducted as described above.

### Statistical analysis

For the pooled nymphs, we estimated prevalence at the individual level using the exact method of Hauck, assuming perfect sensitivity and specificity of our pathogen detection methods [23]. Hauck noted a one-to-one relationship between individual prevalence levels, π, and the prevalence of positive pools, *P*. A point estimate for the prevalence rate can thus be obtained from the pool positive rate by π = 1-(1-P)^1/*k*^ where *k* is the number of nymphs or adults per pool. Exact 95% confidence intervals were then obtained by assuming a binomial distribution for the number of positive pools [24]. For the adults, as each pool only contained two individuals, we calculated the individual prevalence intervals corresponding to prevalence if 2/2 individuals from the positive pools were positive and prevalence if 1/2 individuals from the positive pools were positive.

## Results

### Inventory of medically important bacterial RNA content in ticks

**Figure 1:**
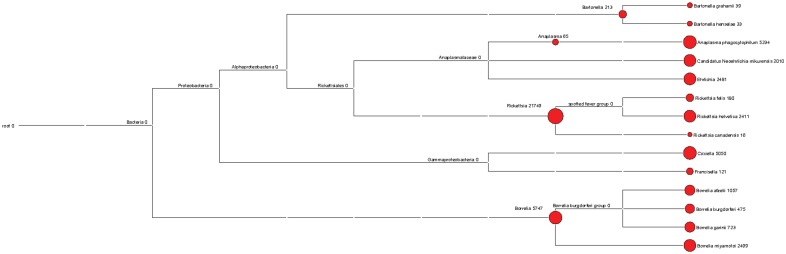
Megan4-generated taxonomy assignment and read numbers based on NCBI reference sequences. Each node is labeled with a taxon, and the number of reads assigned to this taxon.

Bacterial content was organized into genera or putative species by comparison with the closest homolog (Figure 1). Species were only allocated unambiguous assignment to one specific species when either the NGS-derived contig identity percentage was ≥95% compared to a specific species sequence (and lower when compared to other species); or when NGS-derived contigs matched sequences from one single bacterial species. Following these criteria, all bacterial species unambiguously identified are listed in Table 2.

**Table 2:** List of bacteria unambiguously identified at the species level in ticks, including the number of NGS reads for each species, the corresponding list of identified genes allowing taxonomic affiliation (with% identity) to one specific bacteria, and the closest bacterial species to the unambiguously identified species (with% identity).

Unambiguously identified bacterial species (read number)	Gene hit (Accession number) -% id	Closest species -% id
*Anaplasma phagocytophilum* (5234)	16S RNA (JN990105) - 100%	*A. ovis* - 99%
	23S RNA (AF416766) - 100%	*A. marginale* - 96%
*Bartonella henselae* (33)	dnaB (BH0270) - 100%	*B. tribocorum* - 94%
	dnaJ (BH00660) - 100%	*B. quintana* - 91%
	trkA (BH05860) - 100%	*B. quintana* - 93%
	Phage related protein (BH09270) - 100%	*B. grahamii* - 84%
	Hypothetical protein (BH022330) - 99%	*B. tribocorum* - 85%
	Transketolase - (BH15090) - 99%	*B. quintana* - 87%
*Bartonella grahamii* (39)	Phage tail protein encoding gene (ACS5068.1) - 90%	None
	Adhesin (ACS50528.1) - 75%	None
*Borrelia afzelii* (1057)	Hypothetical protein (CP000395)	*B. garinii* - 96%
*Borrelia garinii* (723)	Hypothetical protein (CP001205) - 100%	*B. afzelii* - 98%
	Hypothetical protein(CP003151) -100%	*B. afzelii* - 98%
*Borrelia burgdorferi* s.s. (475)	23S RNA (BORG23S) - 100%	*B. garinii* - 99%
*Borrelia miyamotoi* (2499)	16S RNA (JF951385) - 100%	Other *Borrelia* sp. <98%
*Candidatus* “Neoehrlichia mikurensis” (2010)	16S RNA (JQ359061) - 100%	*A. phagocytophilum* <97%
Ehrlichia canis* (660)	PhoT family ABC transporter (CP000107) - 75%	None
	23SrRNA - 96%	*E. chaffensis* - 96%
*Rickettsia canadensis* (16)	MraW (CP000409) - 96%	*R. felis* - 93%
	TraD (CP000409) - 97%	*R. amblyommii* - 85%
	16S RNA methyltransferase: (CP003304) - 97%	*R. montanensis* - 88%
*Rickettsia felis* (180)	Plasmid pRF (GQ329881) - 98%	*R. australis* - 91%
	Unknown protein (CP000053) - 95%	*R. australis* - 90%
	AmpC (CP000053.1) - 99%	*R. amblyoma* - 97%
	Sca13 (CP000053) - 96%	*R. slovaca* - 91%
*Rickettsia helvetica* (2411)	Sca1 (AY355363) - 100%	*R. felis* - 91%
	ompB (JN036412.1) - 100%	*R. asiatica* - 94%
	groEL (DQ442911) - 99%	*R. canadensis* - 97%
	sca4 (FJ358501.1) - 100%	*R. tamurae* - 94%

Global analysis of genera mainly associated with medically important tick-borne bacteria: *Anaplasma* spp., *Borrelia* spp., *Coxiella* spp., *Ehrlichia* spp., *Francisella* spp. and *Rickettsia* spp., indicated that among the alpha-proteobacteria family, *Rickettsia* spp. were the most highly represented. Species identified included *R. helvetica*, a member of the Spotted Fever Group of *Rickettsiae*, predicted to be associated with human illness in many European countries [25]; *R. canadensis* isolated from ticks belonging to *Haemaphysalis* spp. in Canada [26], never previously identified in Europe, and involved, on serological evidence of infection, in Rocky Mountain Spotted Fever-like disease and cerebral vasculitis in US patients; and *R. felis*, responsible for Spotted Fever in humans and whose only known vector is the flea [27]. As the number of reads was low for *R. canadensis* (16) and *R. felis* (180), and because the presence of both pathogens in *I. ricinus* was not expected, we performed real-time PCR on DNA extracts corresponding to a single pool of 1450 nymphs, which confirmed the detection of *R. felis* DNA, but not *R. canadensis* indicating that for this bacteria the RNA-seq results is below the threshold of detection. We can thus consider that this bacteria is not present in our samples

Among the Anaplasmataceae family, we identified sequences belonging to A. phagocytophilum, the agent of human granulocytosis [25] and “*candidatus* Neoehrlichia mikurensis” a new tick-borne pathogen discovered in 2004. The first human case of “*candidatus* N. mikurensis” infection was reported in a Swedish patient in 2010, but has never been linked to human disease in France thus far [28]. The presence of DNA from this bacterium was confirmed by real-time PCR in the pool of 1450 nymphs. In 2012, we had previously identified this bacterium for the first time in France, in bank voles from North Eastern France [29]. We also identified species belonging to the Ehrlichia genus. NGS sequence analysis combined with specific real-time PCR confirmation facilitated the detection of E. canis, the agent of canine ehrlichiosis, known to be transmitted by the dark tick Rhipicephalus sanguineus [30] (Table 2).

Among the *Bartonella* spp. sequences, the cat-specific *B. henselae* was clearly identified, as well as sequences related to the rodent-specific *B. grahamii* (Table 2). Both of these species are known to be mainly flea-borne zoonotic human pathogens but have also been previously detected in European ticks [31]–[33].

We also detected RNA sequences related to gamma-proteobacteria belonging to *Coxiella* spp. and *Francisella* spp. For these genera, the low number of reads prevented the identification of these bacteria at the species level. We failed to detect *Coxiella burnetti* or *Francisella tularensis* DNA within ticks by real-time PCR, these two species being the most common tick-borne pathogens of each genera.

As expected for ticks collected in Alsace, a French region with high rates of Lyme disease, we also identified *B. afzelii*, *B. garinii*, and *B. burgdorferi s.s.*, the three human pathogenic species known to circulate in France [34]. Interestingly, we also detected sequences from *B. miyamotoi*, which is responsible for relapsing fever in Russia, and which was recently detected in the USA [35]. The presence of *B. miyamotoi* DNA was confirmed by real-time PCR. Even though sequences from this bacteria have previously been amplified from *I. ricinus* in Sweden, France and Slovakia [21], [36], it has never thus far been associated with disease in Europe.

### Prevalence of unexpected human pathogen DNA in ticks

Considering their potential importance to human health and disease, we estimated the prevalence of “*candidatus* Neoehrlichia mikurensis”, *B. miyamotoi*, and *R. felis* microorganisms. Among the 58 tested pools of nymph DNA (each pool containing 25 nymphs), 19 were positive for “*candidatus* Neoehrlichia mikurensis” and 25 for *B. miyamotoi*. By estimating the individual prevalence of nymphs using the exact method of Hauck, we found a prevalence of 2.2% (1.4–3.3) for *Borrelia miyamotoi* and 1.6% (0.9–2.5) for “*candidatus* Neoehrlichia mikurensis”. For adults, we analyzed 31 pools, each containing two adults. We identified two positive pools for “*candidatus* Neoehrlichia mikurensis” which represented an individual prevalence of between 3.2% and 6.4%. All tested adult pools were negative for *B. miyamotoi*. For *R. felis*, we identified one positive pool of nymphs among the 58 tested (estimated prevalence of 0.09%), but none within the adult pools.

## Discussion

We have used a whole transcriptome analysis of ticks RNA to generate a global picture of tick-borne bacteria and validate an NGS strategy, by identifying bacteria at the RNA level. It has been recently shown that metagenomic 16S rDNA Illumina tags are a powerful alternative to 16S DNA amplicon sequencing[37]. This paper extends this approach by using wide transcriptome analysis, including but not limited to 16S and 23 S RNAs. We have identified bacterial species known to be transmitted by *I. ricinus* and causing disease in France or Europe: *Rickettsia helvetica*; *Anaplasma phagocytophilum*; *Borrelia garinii*, *B. afzelii, B. burgdorferi s.s.* and “*candidatus* Neoehrlichia mikurensis”. Considering “*candidatus* Neoehrlichia mikurensis”, has never been identified in ticks from France, we calculated an estimated prevalence of 1.6% in nymphs and from between 3.2 to 6.4% in adults, rates similar to those found elsewhere in Europe [3]. “*candidatus* Neoehrlichia mikurensis” infection has also been detected in humans from north-eastern China, and field surveys showed that 1.6% of ticks and 3.8% of rodents collected from patient residences were also infected [38], [39]. Although corresponding human or animal disease has not yet been reported in France, the prevalence of positive rodents and ticks is similar to levels found in China. In addition, the infected French bank vole (*Myodes glareolus*) and ticks are located in close proximity to human dwellings, suggesting that “*candidatus* Neoehrlichia mikurensis” is very likely to pose risks to public health in France. However, to date, the diagnosis of “*candidatus* Neoehrlichia mikurensis” infection has solely relied on PCR amplification of bacterial DNA. Therefore, the absence of serological tests combined with physicians' lack of information relating to these bacteria, makes diagnosis particularly difficult. Thorough surveillance and improved diagnostic tools are required to provide more insight into the relevance of *Candidatus* N. mikurensis to public health.

In addition to the expected species, our study generated evidence of other unanticipated bacterial species. For example, *B. miyamotoii*, transmitted by *Ixodes* spp., and causing recurrent fever, has not previously been associated with disease in Europe [35]. *B. miyamotoi* was isolated for the first time in Japan in 1995 from *I. persulcatus* ticks as well as from the blood of *Apodemus argentus* mice [40], [41]. In Europe, *B. miyamotoi* was detected in ticks from Sweden, Germany, and very recently, from Estonia [36], [42], [43]. In addition to *A. argentus* mice, it has been shown that other rodents such as white-footed mice (*Peromyscus leucopus*) may serve as host reservoirs for *B. miyamotoi* [44]. In our study *B. miyamotoi* was estimated to occur at a mean prevalence rate of 2.2% in nymphs, a rate close to those obtained from different European countries, which fluctuates from 0.4% in Estonia to 3.5% in Germany [35], [36], [43], [45]. Some symptoms caused by this particular *Borrelia* sp., such as recurring high fever, are very different from the skin rash and joint pain typical of Lyme disease. Identifying the RNA of this bacterium in French *I. ricinus* for the second time strongly suggests that the disease could exist in this country. However, in the absence of specific tests, it is impossible to know whether the disease is indeed present in France (or in Europe), and if it has the potential to cause serious long-term damage, as can result from untreated Lyme disease.

We also detected known pathogens which are either normally transmitted by other tick species, or by vectors other than ticks, such as for the *B. henselae*, *B. grahamii* and *R. felis* microorganisms. *B. henselae* and *R. felis* are typically transmitted from cat to cat via fleas, with human contamination arising from cat or flea bites. Establishing ticks as vectors of *B. henselae* has been fiercely debated for many years, even given the recent demonstration of the competence of *I. ricinus* to host *B. henselae* [46]. To our knowledge, our data present the first characterization of *R. felis* RNA in ticks. However even though we have confirmed the presence of RNA and DNA from *R. felis* in these ticks by real time-PCR with specific primers, the detection of *R.felis* DNA in only one pool of nymphs strongly suggests, if it exists, an anecdotic role for *I. ricinus* in *R. felis* circulation.

Detecting RNA of unexpected pathogens in *Ixodes* ticks indicates that these microorganisms are viable and actively replicating, however, it does not prove that transmission by *I. ricinus* actually occurs. Competence studies of *I. ricinus* for all detected pathogens should be experimentally clarified in the future before reclassifying them as tick-borne pathogens. Although further research is thus required to confirm the role of *I. ricinus* in transmitting these pathogens, the data obtained with NGS has proven that this method has enormous potential to detect the unexpected, and provides an excellent means for surveying pathogen occurrence in disease vectors from particular localities.
